# A Tiny Matched Filter-Based CNN for Inter-Patient ECG Classification and Arrhythmia Detection at the Edge

**DOI:** 10.3390/s23031365

**Published:** 2023-01-26

**Authors:** Mohammed M. Farag

**Affiliations:** 1 Electrical Engineering Department, College of Engineering, King Faisal University, Al-Ahsa 31982, Saudi Arabia; mfarag@kfu.edu.sa; 2 Engineering Department, Faculty of Engineering, Alexandria University, Alexandria 5424041, Egypt; mmorsy@alexu.edu.eg

**Keywords:** machine learning, convolutional neural network, interpretable neural network, matched filter, electrocardiogram

## Abstract

Automated electrocardiogram (ECG) classification using machine learning (ML) is extensively utilized for arrhythmia detection. Contemporary ML algorithms are typically deployed on the cloud, which may not always meet the availability and privacy requirements of ECG monitoring. Edge inference is an emerging alternative that overcomes the concerns of cloud inference; however, it poses new challenges due to the demanding computational requirements of modern ML algorithms and the tight constraints of edge devices. In this work, we propose a tiny convolutional neural network (CNN) classifier for real-time monitoring of ECG at the edge with the aid of the matched filter (MF) theory. The MIT-BIH dataset with inter-patient division is used for model training and testing. The model generalization capability is validated on the INCART, QT, and PTB diagnostic databases, and the model performance in the presence of noise is experimentally analyzed. The proposed classifier can achieve average accuracy, sensitivity, and F1 scores of 98.18%, 91.90%, and 92.17%, respectively. The sensitivity of detecting supraventricular and ventricular ectopic beats (SVEB and VEB) is 85.3% and 96.34%, respectively. The model is 15 KB in size, with an average inference time of less than 1 ms. The proposed model achieves superior classification and real-time performance results compared to the state-of-the-art ECG classifiers while minimizing the model complexity. The proposed classifier can be readily deployed on a wide range of resource-constrained edge devices for arrhythmia monitoring, which can save millions of cardiovascular disease patients.

## 1. Introduction

According to the World Health Organization [1], cardiovascular disorders caused by chronic ventricular arrhythmias are the primary cause of mortality worldwide. The complexities of arrhythmias and their clinical and mechanical interdependence result in frequent cross-classifications and misdiagnoses using visual criteria. Electrocardiography is still the most commonly used method for arrhythmia diagnosis due to its simplicity, efficiency, and low cost [2]. An electrocardiogram (ECG) is a record of the electric waves generated during heart activity that provides sensitive information about cardiac function. Automated classification of ECG is extensively utilized for arrhythmia detection [3]. ECG classification methods using Artificial Intelligence (AI), Machine Learning (ML), and Deep Learning (DL) techniques have achieved impressive results during the last decade [4].

Continuous monitoring of the ECG activity accompanied by automated arrhythmia detection in real-time enables early identification of sudden heart arrhythmias, which can save millions of lives from chronic cardiovascular diseases. For detecting arrhythmia in real-time, a single-lead ECG wearable can capture the ECG signal and deliver it to a cloud machine running an ECG classification model. Nowadays, such an approach can be easily employed owing to recent advances in sensor technology, automatic ECG classification methods, and cloud services [5].

Hannun et al. [6] proposed a deep neural network (DNN) to classify heartbeat signals using a single-lead ambulatory ECG monitoring device. With specificity fixed at the average cardiologist’s specificity, the sensitivity of the proposed DNN outperformed the average cardiologist’s sensitivity for all rhythm classes. These findings reveal that an end-to-end DL-based arrhythmia detection approach can classify a broad spectrum of distinct arrhythmias from single-lead ECGs with accuracy surpassing that of cardiologists. By appropriately selecting the most urgent conditions, this strategy might minimize the rate of misdiagnosed automated ECG readings and enhance the efficiency of expert-human ECG interpretation.

Nowadays, cloud inference, in which model predictions are computed remotely on a cloud server, is the dominant deployment approach of modern ML and DL models [5]. The main concerns of cloud inference are patient privacy, internet latency, sensor connectivity, and service availability, all of which prevent the wide application of automated arrhythmia detection. Edge inference, in which an edge device is used to compute model predictions locally, is an emerging alternative that addresses the concerns of cloud inference.

In this work, we propose inter-patient ECG classification and arrhythmia detection at the edge. Instead of depending on a cloud service to detect arrhythmias, an edge microcontroller device is used to gather and classify ECG data in real-time and notify the patient to take precautions. However, edge deployment of AI models is challenging due to the computational complexity of contemporary AI algorithms and the limited resources of edge devices. Furthermore, this challenge is augmented by the stringent accuracy requirements of arrhythmia detection. Moreover, the inter-patient classification of ECG signals is a challenging ML problem because the training and testing sets come from different sources with inherited inter-individual variations. In response, the matched filter (MF) interpretation of the convolutional neural network (CNN) presented in our previous work [7] is exploited to address these challenges and develop a tiny ECG classifier for edge inference. MFs are optimal filters for signal detection in the presence of noise, and their operation is tightly linked to CNNs [8].

The MIT-BIH dataset with inter-patient division is used for model training and testing [9]. The CNN classifier is carefully designed to meet the application accuracy requirements and edge device computational constraints. The raw ECG signal is fed directly to the CNN classifier without pre-processing or feature engineering procedures to minimize the computational load on the edge device. The MF-based CNN model is optimized for edge inference by applying state-of-the-art weight quantization and pruning methods. The model is extensively tested and benchmarked on a cloud machine and a raspberry-pi edge microcontroller. The model’s generalization capability is validated on the INCART, QT, and PTB diagnostic databases, and the model performance in the presence of noise is experimentally analyzed. Testing results show that the proposed model achieves superior classification and real-time performance results compared to the state-of-the-art ECG classification methods. Contributions of this work include:

- Exploiting the MF interpretation of CNNs to develop a tiny ECG classifier ready for edge deployment.
- Investigate using the first derivative of the ECG signal as an input feature for ECG classification and demonstrate its superiority to using the raw ECG signal.
- Extensively testing the ECG classifier on a raspberry-pi edge device, reporting its performance and benchmarking results, comparing our work to recent state-of-the-art inter-patient ECG classification methods, and showing its competency.
- Validating the model’s generalization capability on several recognized ECG datasets and analyzing the model performance in the presence of noise.

The remainder of this paper is organized as follows. In Section 2, ECG classification-related work is presented. An overview and preparation of the MIT-BIH dataset for ML are presented in Section 3. The proposed MF-based CNN classifier is advanced in Section 4. Methods and tools used in this work are introduced in Section 5. Model testing results on the cloud and edge machines, accompanied by a comparison with state-of-the-art ECG classification methods, are presented in Section 6. Analysis of model generalization ability and performance in the presence of noise is also presented in Section 6. Conclusions and future work are portrayed in Section 7.

## 2. Related Work

**Figure 1:**
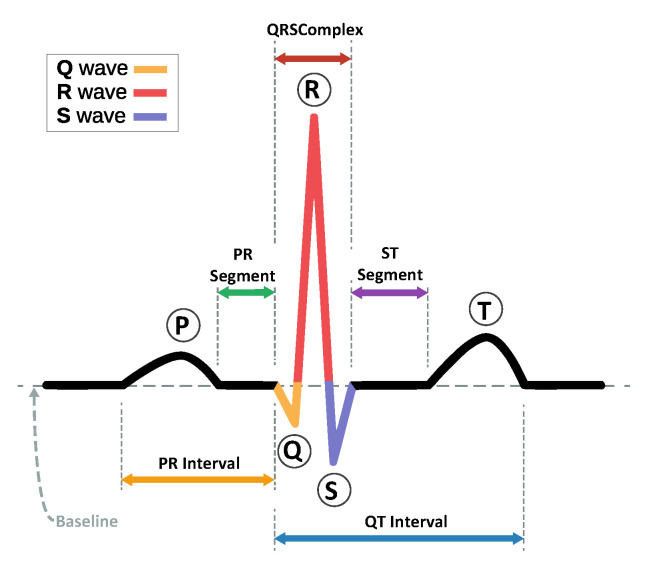
ECG of a heart in normal sinus rhythm.

A typical ECG waveform consists of a P-wave, a QRS complex wave, and a T-wave, as illustrated in Figure 1, which reflect the electrical activities of the depolarization and repolarization processes of the atria and ventricle [2]. Each heartbeat contains a series of deflections away from the baseline on the ECG that reflect the time evolution of the heart’s electrical activity. The P-wave is a slight defection caused by atrial depolarization; the Q, R, and S waves are known as the QRS-complex, which is the largest-amplitude portion of the ECG, caused by ventral depolarization; and the T-wave is caused by ventral polarization. Up to 12 separate leads can be used to measure an ECG, in which each lead illustrates the heart’s electrical activity from a particular angle across the body. The normal heart rhythm is called sinus rhythm, in which the triggering impulses propagate throughout the heart’s four chambers in a coordinated manner. Changes in the normal ECG pattern occur in numerous cardiac abnormalities called arrhythmias, which occur due to changes in the heart’s normal sequence of electrical impulses. The ECG is the most effective tool to spot and identify several types of arrhythmias.

The MIT-BIH arrhythmia database is the most acknowledged dataset in the academic literature [2]. The database contains 48 half-hour excerpts of two-channel ambulatory ECG recordings obtained from 47 subjects [10,11]. A total of 15 beat annotations denoting several arrhythmias are assigned to the R-peaks of the ECG heartbeats. Three protocols are proposed for partitioning the MIT-BIH dataset into training and testing sets: intra-patient, inter-patient, and random division schemes [2]. In the random division scheme, the whole dataset is randomly divided into training, and testing sets such that both sets keep the same distribution, which meets the principles of ML. In the intra-patient division scheme, heartbeat segments from the same patient record are used for training and testing, where a subset of the ECG beats is used for training, and the remaining part is used for testing. In the inter-patient division scheme, the training and testing sets are split by record number, so heartbeats within each set come from distinct subjects. The inter-patient division scheme is the most realistic approach as it resembles real situations in which a model is trained on data collected from a set of individuals and applied to another set. However, studies that followed the inter-patient division scheme reported great difficulty in obtaining promising results for the heartbeat arrhythmia classes Supraventricular Ectopic Beat (SVEB) and Ventricular Ectopic Beat (VEB) [2]. In this work, we will follow the inter-patient division scheme, aiming to enhance the detection results of arrhythmic minority classes.

Several works have been presented in the last decade addressing automatic ECG-based heartbeat classification methods. Sahoo et al. [4] presented a survey of ML approaches to detect cardiac arrhythmias in ECG signals. According to this survey, deep learning techniques are more efficient than standard classifiers in terms of accuracy and computational complexity, which are essential in real-time applications. Luz et al. [2] surveyed state-of-the-art ECG-based automated heartbeat classification methods, databases, and evaluation standards. The most prevalent ECG classification methods in the literature are DNNs and support vector machines (SVMs). In this section, we will survey related ECG classification methods, emphasizing those that adopt the inter-patient division scheme. In Section 6, we will compare the proposed model with related models presented in this section at the level of classification and real-time performance results.

Ebrahimi et al. [12] presented a comprehensive review of recent DNN methods for ECG classification. According to this review, the gated recurrent unit (GRU), long short-term memory (LSTM), CNN, and LSTM DNN models showed outstanding results for the correct classification of atrial fibrillation (AF), SVEB, and VEB, respectively. However, recurrent neural networks (RNNs) have several limitations, including limited generalization capability for smaller datasets, noise effects on classification accuracy, and high-computational costs limiting their applicability to edge inference.

Zhang et al. [13] introduced a CNN-based adversarial DL model for inter-patient heartbeat classification comprising an encoder, classifier, and adversary networks. ECG heartbeat segments and normalized local and global RR intervals are the features fed to the classifier as separate channels. The average RR intervals are computed for the whole patient record, which does not consider causality for real-time implementations. The convolutional encoder is used to learn representations from the extracted features. Then, these representations are fed separately into the classifier decoder and the adversary network to classify heartbeats and subject IDs, respectively. The complexity of the proposed model limits its application to edge computing.

Wang et al. [14] proposed an inter-patient ECG classifier model based on continuous wavelet transform (CWT) and CNNs that can be used as a clinical auxiliary diagnostic tool. ECG signals are pre-processed for noise and baseline wandering removal using two consecutive median filters. Several RR interval features have been extracted, including post-, pre-, local-, and ratio-RR intervals. CWT with the Mexican Hat mother wavelet is used to compute the time-frequency scalogram of the ECG heartbeat segments, which are then fed as images to a 2D CNN along with the RR intervals for beat classification. The CWT pre-processing procedure incurs an additional computational cost for the classifier, limiting its applicability to edge inference.

Mondéjar-Guerra et al. [15] advanced a method for automatic ECG classification based on a combination of multiple SVMs. Two consecutive median filters are used for baseline wandering removal. The method relies on the time intervals between the subsequent beats and their morphology for ECG characterization. Different features based on the discrete wavelet transform (DWT), local binary patterns (LBP), higher-order statistics (HOS), and several amplitude values are extracted. Other morphological features, such as normalized RR intervals and signal peaks, are also used. Instead of concatenating all these features to feed a single SVM model, specific SVM models are trained for each type of feature, and the final prediction is obtained by combining the decisions of the different models with the product, sum, and majority rules. The applicability of this model to edge inference is limited by the use of multiple pre-processing steps and SVM models, which increase the model’s complexity.

Raj and Ray [16] advanced a personalized monitoring system for detecting heart arrhythmias in real-time. Discrete Orthogonal Stockwell Transform (DOST) is proposed for time-frequency feature extraction, and the Artificial Bee Colony (ABC) optimized twin least-square support vector machine (LSTSVM) algorithm is used for signal classification. Two median filters and a 12-tap finite impulse response (FIR) low-pass filter (LPF) are implemented for baseline wandering removal, high-pass noise reduction, and power-line interference filtering. DOST is computed for an ECG signal by applying the Fast Fourier Transform (FFT), windowing, and inverse FFT (IFFT) to calculate a set of coefficients representing the time-frequency morphological features of the signal. The ABC-LSTSVM is a reduced-complexity SVM algorithm tailored to fit embedded device constraints. The proposed platform is prototyped on an ARM9 embedded kit and experimentally validated on the MIT-BIH database for the intra- and inter-patient division schemes. Although the proposed platform is recommended for hospitals to analyze long-term ECG recordings, the model size, memory usage, and real-time performance results are not provided.

Garcia et al. [17] presented inter-patient ECG classification with temporal vectorcardiogram (TVCG) along with a complex network for feature extraction optimized by a particle swarm optimization (PSO) algorithm. The VCG is a two-dimensional representation of the ECG that uses the signal from two distinct leads. The VCG employs time as a third dimension and each lead as an axis of a 2D display. Several morphological and interval features, including the RR intervals, are extracted. Additionally, DWT and autocorrelation extract time-frequency and signal coherence features. A subset of these features is optimized and selected by the PSO algorithm and fed to an SVM classifier. The high computational complexity of this method limits its applicability to edge inference.

Chen et al. [18] introduced an ECG classification method based on a combination of projected and dynamic features. DWT is used for signal denoising. Projected features are derived from a random projection matrix, in which each column is normalized, and each row is transformed by discrete cosine transform (DCT). Additionally, three weighted RR intervals are used as dynamic features. An SVM classifier is used for ECG classification. The used pre-processing steps and SVM classifier are not suitable for edge computing.

Zhang et al. [19] proposed a feature selection method that consists of a one-versus-one (OvO) feature ranking stage and a feature search stage wrapped in the same OvO-rule SVM binary classifier. Several inter- and intra-beat intervals, morphological amplitudes, areas, and distances are extracted as heartbeat features. The features of two leads are extracted and fed to a binary OVO SVM classifier to select the effective subsets of characteristics and classify the ECG signal by combining the classifiers.

Lin and Yang [20] advanced an ECG classifier based on normalized RR intervals and morphological features. Normalized post-, pre-, local-, and global-RR intervals, zero-crossings, and peak positions are extracted and fed to the classifier. The average RR intervals are computed for the whole patient record, which does not consider causality for real-time implementations. Morphological features are extracted using DWT, autocorrelation, and linear predictive modeling (LPM). The Linear Discriminant Classification (LDC) method combines the extracted RR intervals and morphological features and performs ECG classification.

Bansal et al. [21] presented ECG template-based classification of cardiac arrhythmia to automatically classify normal heartbeats versus premature ventricular contraction (PVC) beats on portable devices. Normal and PVC average beat templates have been extracted for all patient records with MLII readings from the MIT-BIH dataset. Dynamic time warping (DTW) is used for feature extraction by finding the similarity score between the classified heartbeat and the template heartbeats, which is then fed to a K-nearest neighbor (KNN) classifier. This work considered using average heartbeat templates for arrhythmia detection, which is shared with our work, yet the achieved classification results fall behind state-of-the-art rivals by a significant margin.

In our previous work [5], an FIR-based interpretation of the Conv1D layer is presented and exploited to develop a self-contained short-time Fourier transform (STFT)-based CNN ECG classifier. The Conv1D layer kernels are designed as a filter bank for extracting the time-frequency spectrogram of the input ECG signal. The Conv1D layer feature maps are reshaped into a 2D heatmap image and then fed to a 2D CNN for classification. The developed model is applied to the intra-patient ECG classification problem and achieves superior classification and real-time performance results compared to the state-of-the-art models. Unfortunately, this model does not achieve comparable results for the inter-patient ECG classification problem. In Ref. [7], we presented the MF interpretation of CNN classifiers with application to human activity recognition. The developed model achieves superb classification performance with significantly reduced complexity compared to related models. The results of this work encouraged us to extend the application of the proposed MF CNN classifier to the ECG classification problem.

In this work, we aim to develop a tiny ECG classifier for real-time monitoring cardiovascular arrhythmias at the edge. This is challenged by the stringent accuracy requirements of the non-trivial inter-patient ECG classification problem and the resource constraints of modern edge devices. Unfortunately, this challenge is not commonly addressed in the ECG classification literature. Other challenges include the dataset imbalance problem and the inter-patient division scheme recommended for realistic model evaluation. Furthermore, there is still room for enhancing the inter-patient ECG classification results, especially in detecting the arrhythmic ventricular minority classes.

## 3. Dataset Preparation and Feature Selection

In this work, the MIT-BIH arrhythmia database with the inter-patient division scheme is used for model training and testing [10,11]. This database contains 15 beat annotations denoting various categories of normal and arrhythmic heartbeats collected from different individuals. According to the standard developed by the Association for the Advancement of Medical Instrumentation (AAMI) [22], 17 arrhythmia categories are mapped into five essential groups or super-classes. The AAMI standard emphasizes the problem of distinguishing ventricular from non-ventricular ectopic beats. In this work, we followed the AAMI standard, which is commonly used in the literature [2,3,4] to standardize the evaluation process considering the clinical point of view and AAMI recommendations and ensure a fair comparison with the related work. Table 1 shows the annotated arrhythmia classes in the MIT-BIH dataset. As demonstrated in Table 1, annotations in the MIT-BIH dataset are mapped to five distinct beat types serving as dataset labels following the AAMI standard. Eventually, the total number of classes will be the five AAMI super-classes, each comprising several sub-classes representing the MIT-BIH arrhythmia types.

The MIT-BIH database signals were extracted from Holter recordings and filtered to limit analog-to-digital converter (ADC) saturation using a band-pass filter (BPF) from 0.1 to 100 Hz [11]. Each ECG record in the MIT–BIH arrhythmia database includes two leads originating from different electrodes; the most typical leads are the modified limb lead II (MLII), and chest lead V1. The MLII is the most accessible lead for ECG data acquisition and arrhythmia detection as it highlights various segments within the heartbeat [2]. During model training, the raw MLII lead signals will be fed directly to the classifier as they are extracted from the MIT-BIH database without pre-processing. For edge deployment, a pre-processing baseline wandering and noise removal stage will be implemented using a low-cost median filter and an FIR LPF as instructed by [14,15,16,17,20], or moved to the ECG analog front-end to reduce the computation load on the edge device.

To prepare the MIT-BIH database signals for ML, ECG signals are downsampled to 128 samples/s. ECG signals from selected records are segmented on a beat-by-beat basis by filtering out non-beat annotations from the database and extracting 0.5-s segments (64 samples) centered at the annotated R-peak. The selected sampling frequency and segment duration are sufficient to represent the heartbeat morphology while reducing the model input size and computational complexity. Heartbeat segments with intervals less than 0.5 s are edge-padded to unify the segment length, which is required for the DNN model input.

RR intervals are the main features for ECG classification since many arrhythmic ECG beats have a noticeable change in the RR intervals. However, using the absolute values of the RR intervals can degrade the classification accuracy due to the inter-individual variation of the regular heartbeat rate of different individuals. Using dynamic normalized RR-intervals significantly enhances the classification results [20]. Dynamic local and global means of the RR intervals are used to normalize the pre- and post-RR intervals. The dynamic local RR interval mean is collected by applying a moving average operator to the last 80 RR intervals (around 1 min for standard heartbeat rate), while the dynamic global RR interval mean is collected by averaging the last 400 RR intervals (around 5 min of normal heartbeat rate).

The global and local RR intervals are selected by developing an XGBoost model [23] for ECG classification using only the dynamic normalized RR intervals and conducting an iterative search to find the best dynamic local and global intervals that maximize the classification accuracy. The tunable search parameters are the moving average window sizes of the local and global RR intervals. XGBoost (eXtreme Gradient Boosting) is a popular and efficient supervised learning algorithm that accurately predicts a target variable by combining an ensemble of estimates from a set of more superficial and weaker models. Using only the local and global RR intervals of 80 and 400 beats, the XGBoost model can achieve a classification accuracy of 93% with an average recall and precision scores of 75% and 58%, respectively, on the inter-patient dataset division scheme described next. More specifically, the XGBoost classifier achieves recall, precision, and F1 scores of 97%, 97%, 97%; 60%, 36%, 45%; and 58%, 75%, 65% for the “N”, “SVEB”, and “VEB” classes, respectively. This classifier outperforms many existing inter-patient ECG classifiers presented in the literature; however, these results are not satisfactory for arrhythmia detection.

Causality and implementation feasibility have been considered in extracting the local and global means of the RR intervals, where only the past RR intervals are needed for moving average calculations. The preceding and subsequent RR peak intervals have been extracted from the MIT-BIH dataset annotations and normalized to the local and global RR interval means, which are calculated only using the past values of RR intervals. The dynamic normalized RR intervals are fed as input features to the proposed classifier model. The proposed interval extraction method is straightforward and can be easily implemented on the edge device using a simple peak detector and an averaging algorithm with 400 memory locations for storing the past RR interval values. It should be indicated that the inter-patient ECG classification problem is susceptible to the RR interval input features, which should be carefully selected as their improper selection would lead to high variance between the training and testing results due to model overfitting and inter-individual variations. An example of improper selection of the RR interval features is feeding the absolute values of the pre- and post-RR intervals rather than the normalized intervals to the classification model, directly affecting the classification performance on the testing set.

In this work, the MIT-BIH dataset is partitioned according to the inter-patient division scheme proposed by Chazal et al. [9]. Most inter-patient ECG classification works in the literature [13,14,15,16,17,18,23,24] apply the same division scheme, which facilitates conducting fair comparison and evaluation. Table 2 shows the records used in training and testing sets DS1 and DS2 and the number of heartbeats per AAMI class. The inter-patient division scheme is more realistic since it resembles the actual scenarios in which the classifier is trained using data collected from a group of patients and applied to a different group. However, the inter-patient division scheme is much more challenging for ML because the training and testing datasets do not have the same distribution and due to inter-individual variations between the training and testing dataset examples. Therefore, the inter-patient ECG classifiers suffer from performance degradation compared to the counterpart classifiers of intra-patient and random division schemes.

## 4. Matched Filter-Based Convolutional Neural Network Classifier

### 4.1. Matched Filter Interpretation of the Convolutional Neural Network

A CNN classifier is a DL model comprising a hierarchical stack of convolutional, pooling, and fully connected (FC) dense layers. The convolutional and pooling layers are used for feature extraction and data dimensionality reduction. Features extracted by these layers are then fed to a stack of FC layers for classification. A 1D convolutional (Conv1D) layer comprises multiple 1D filters, also called kernels, which are correlated with the input sequence to produce output feature maps [25]. A bias parameter is used to fine-tune the kernel output for improved performance. The number of strides parameter determines the shift amount, and the dilation rate controls the spacing between the kernel points. For a standard Conv1D layer with the stride length and dilation rate set to 1, the layer output is defined as follows:

(1) $$y_{c_{out}}\left[n\right]=b_{c_{out}}+\sum_{i=1}^{C_{in}}w_{c_{out}}^{i}\left[n\right]⋆x_{i}\left[n\right]$$

where y[n] is the layer output, *b* is the bias, w[n] is the kernel weight vector, x[n] is the layer input, $c_{out}$ denotes the output channel, and $C_{in}$ denotes the total number of input channels.

The last equation shows the operation of a typical multi-input, multi-output Conv1D layer, where the output channel is computed as the sum of the correlation between the input channels and various channel-specific kernels. There are Conv1D layer variants in which each input channel is correlated with a different kernel, independent of other channels. For a Conv1D layer with a single input channel, the layer output is computed as the correlation between the input channel and multiple CL kernels, producing different output channels or feature maps. Herein, we will focus on the single-input, multi-output Conv1D layer for the single-lead ECG signal.

**Figure 2:**
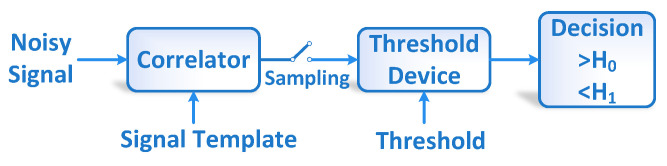
Block diagram of the matched filter receiver.

Despite their prevalence, CNNs are employed as “black box” models because their internal operations and decision mechanisms are not explicitly understood [8]. The MF theory has been exploited to explain the CNN operation in the time domain [7]. An MF is an optimal filter for signal detection in the presence of noise. Figure 2 depicts the block diagram of the MF receiver. The MF decision is computed by correlating a template of the signal to be detected with the unknown signal and comparing the maximum correlation output to a pre-set threshold. The MF correlator output is defined as:

(2) $$y\left[n\right]=x\left[n\right]⋆h\left[n\right]=\sum_{i=1}^{N}x\left[i\right]h\left[i-n\right]$$

where y[n] is the correlator output, x[n] is the input signal, and h[n] is the template signal.

Comparing (1) and (2) illustrates that a Conv1D kernel can implement the MF correlation operation with the template signal assigned to the kernel weights. The shifting operation is performed by sliding the kernel for all values of *n* (for the number of Conv1D strides is set to 1). A GlobalMaxPooling (GMP) layer is instantiated to sample the maximum output of the Conv1D layer and perform the operation of the MF sampling device. A nonlinear activation function such as ReLU or Tanh is used to implement the thresholding operations. This stack of Conv1D, nonlinear activation, and GMP layers work together as multiple MFs with templates $h_{i}\left[n\right]=w_{i}\left[n\right]$, where *i* denotes the ith kernel of the Conv1D layer. An FC layer can be instantiated and trained to map the MF outputs to the relevant class outputs of the classifier model using its weighted sum functionality.

### 4.2. Matched Filter-Based ECG Classifier

**Figure 3:**
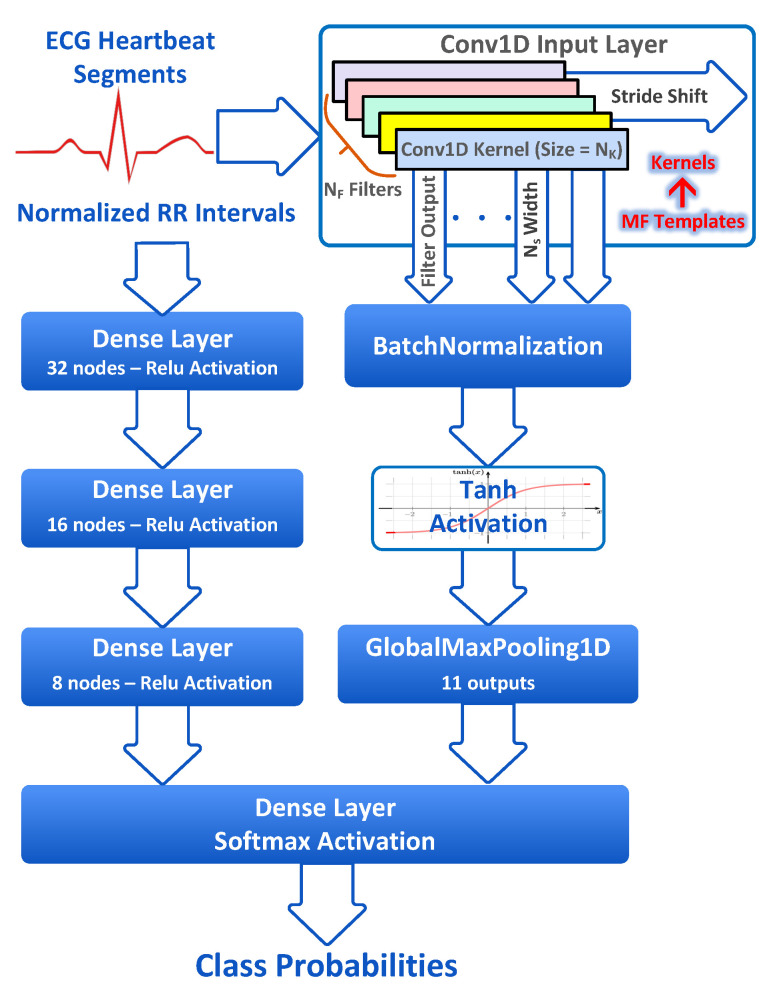
The match filter-based CNN ECG classifier model.

In this work, we exploit the MF interpretation of CNNs to develop an ECG classifier with pre-assigned kernel weights representing the templates of various ECG classes. Figure 3 shows the MF-based CNN classifier model. For the MIT-BIH dataset, MF kernels are computed by averaging all heartbeat segments belonging to each MIT-BIH ECG sub-class presented in Table 1 for the training dataset DS1. A total of 13 MFs, each of a 64-sample length (the length of ECG segments), are extracted and assigned to a Conv1D layer kernel with the number of filters $N_{F}=13$ and kernel size $N_{K}=64$. The Conv1D layer is followed by BatchNormalization (BN) and GMP layers. A BN is a regularization layer for reducing the covariate shift and instability in the layer activation distributions and mitigating the vanishing gradient problem in model training. The GMP layer outputs the maximum values of feature maps. Concurrently, the dynamic normalized RR intervals are fed to a stack of FC layers. For feature fusion, the outputs of the GMP layer and RR interval stacks are concatenated and fed to the FC output layer with Softmax activation to produce class probabilities.

The MF CNN classifier operation is described as follows: The Conv1D layer correlates an input heartbeat segment with the pre-assigned ECG class templates. For an input heartbeat segment, a single kernel will compute the autocorrelation between the signal and the associated template, while the remaining kernels will compute the cross-correlation between the signal and the unmatched kernel templates. The GMP layer will select the maximum output of all Conv1D filters fed to the FC output layer. Weights of the FC output layer and the RR interval stack will be learned during model training to minimize discrepancies between the predicted and ground truth labels. Uncorrelated signals annotating various arrhythmia classes will be optimally classified using the MF-based CNN model; however, inter-individual variations between the training and testing sets will affect the model performance.

The complexity of the proposed model is minimized by employing a single Conv1D layer with a large receptive field, alleviating the need for deeper hierarchical models. The number of model-trainable parameters is minimized by pre-assigning the Conv1D kernels, which reduces the model training time and facilitates model training on small datasets. The MF-based CNN classifier meets the accuracy requirements of the inter-patient ECG classification problem and the computational constraints of edge inference, enabling real-time arrhythmia detection.

## 5. Methods and Tools

### 5.1. Handling Class Imbalance

The MIT-BIH dataset is highly imbalanced, with a majority class “N” to minority class “Q” ratio of 6008, and the percentage of normal ECG beats to the total number of beats is 89.47% for the inter-patient division scheme as shown in Table 2. Addressing class imbalance using classic ML approaches has been intensively investigated over the last decade. Methods for handling class imbalance are categorized into data-level methods, algorithm-level approaches, and hybrid techniques [26]. Data-level approaches for addressing class imbalance include undersampling and oversampling the dataset examples via repetition or synthetic creation of examples. In contrast, algorithm-level approaches handle class imbalance by increasing the weights of the minority class in the model optimization objective function during model training. In this work, the class imbalance problem of the MIT-BIH database is addressed at the algorithmic level by incorporating a class weight parameter to assign higher weights to minority classes according to the class distribution.

On the other hand, classes “F” and “Q” have the least number of examples and are irrelevant to heart arrhythmia. Therefore, several variants of the model with and without the minority classes “F” and “Q” have been investigated to study the effect of eliminating the minority classes on model performance. Three model variants are investigated: all five AAMI classes are involved, four classes are considered (excluding class “Q”), and only the three classes “N”, “SVEB”, and “VEB” are considered. Such an approach is commonly used in the literature [13,14,15].

### 5.2. Extraction of Matched Filter Templates

The model is fed the ECG segments directly. Due to the morphological correlation between some ECG heartbeats belonging to different classes, the first derivative of the heartbeat will be investigated as an input feature to the CNN classifier and compared to the ECG segment feature. The ECG first derivative has been used in previous works for many purposes, including segmentation and QRS detection [27,28], because the first derivative of an ECG heartbeat reveals subtle variations and high-frequency components in the signal, making it a more efficient discriminative feature for the classification task. The ECG’s first derivative is computed by applying the discrete difference operator, which is equivalent to continuous differentiation, to the ECG segment.

MF templates are extracted for all sub-classes in the MIT-BIH dataset and assigned to Conv1D layer kernels. An MF template is computed as the mean of all examples per class in the training set DS1. This procedure results in 13 MF templates of 64 samples each, which will be assigned to the Conv1D layer kernels. This procedure of extracting MF templates is performed once at the training time, and the computed templates will be embedded in the Conv1D layer weights of the model that can be used directly for inference. The difference operator will be applied to the input heartbeat segments at inference time to compute the first derivative fed to the CNN classifier.

**Figure 4:**
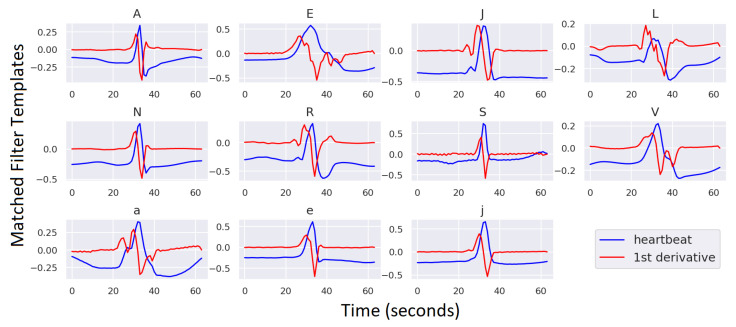
MF templates of the ECG heartbeat and its first derivative for all AAMI classes included in DS1 (excluding the “F” and “Q” classes).

MF templates of the ECG heartbeats and their first derivatives are shown in Figure 4 for all ECG sub-classes in DS1 that are used for model training (excluding “F” and “Q” classes). As evidenced by Figure 4, the first derivatives of the heartbeats have greater diversity and are thus better used to discriminate between different ECG classes than the heartbeat signals. To prove this, we will develop two variant ECG classifier models fed with the raw heartbeats and their derivatives and compare the classification results achieved by each model. We will also develop a CNN model with two input channels for the ECG signal and its derivative, with MF templates assigned for both channels. Figure 4 demonstrates that the non-constant values of the MF templates for both the signal and its derivative are concentrated around the R-peak. This notice indicates that approximately half of the template window (around 16 samples from each edge) can be discarded to reduce the number of Conv1D kernel parameters $N_{K}$ from 64 to 32, reducing the model complexity.

Other options for extracting the MF templates are averaging all heartbeat segments belonging to the same AAMI super-class, which would result in 5 template signals, or computing a template per patient record per sub-class, which would result in 79 template signals. All three options for computing the MF templates have been investigated, and the best classification results were obtained for the option with 13 template signals. This result is expected since averaging over the five AAMI classes would merge heartbeats from different sub-classes included in the super AAMI class and lose the morphology of the sub-class beats, while averaging over patient records would result in the development of personalized heartbeat templates and increase the ambiguity of the classification model.

### 5.3. Workflow and Tools

Keras with the Tensorflow backend is used to train and test the CNN model. TensorFlow is an open-source framework for ML created by Google with an extensive set of tools, libraries, and community resources that enable building and deploying ML-powered applications. Keras is an open-source package that provides a high-level Python interface to the TensorFlow library. Several variants of the ECG classifier model shown in Figure 3 are developed and tested to study the effect of various model parameters. The model variation parameters include the MF template length $N_{K}$ (32 or 64), the number of classes (3, 4, and 5); using the ECG heartbeat segments or their derivatives or both as an input feature; and setting the Conv1D trainable parameter to either “False” or “True” to check the effect of training the Conv1D layer weights on the classification accuracy; for a trainable Conv1D layer, the layer weights are either initialized to the MF templates or initialized to the default initial weights set by the Keras initializer (Glorot uniform initializer).

The categorical cross entropy loss function and Adam optimizer with adaptive learning rate scheduling initialized at 0.001 are used for model training. DS1 is divided by the patient records into training and validation sets to imitate the inter-patient division of DS1 and DS2. Record numbers less than 200 are used for model training, while the remaining are used for model validation. The epochs and batch sizes are set to 500 and 512, respectively, with an early stopping callback to avoid model overfitting. The training was conducted on a workstation featuring 8 CPU cores, 30 GB of RAM, and an NVIDIA QUADRO RTX 5000 GPU. The experiments are repeated ten times for each model variant, and the average classification scores are reported.

Afterward, the TensorFlow lite (TFLite) optimization tools [29,30] and the Google Qkeras library [31] are used for optimizing models with the best scores for edge inference. Quantization algorithms compute and store tensors with bit widths less than the floating-point precision. Instead of the standard 32-bit single-precision floating-point, a quantized model performs operations on tensors with integer or lesser float precision. Quantization yields more compact model representations, lower memory footprints, quicker inference, and less-demanding processing needs at the expense of insignificant accuracy loss. Quantization-aware training (QAT) [32,33] and post-training quantization (PTQ) [34] methods are applied to create the TFLite models. QAT results in less reduction in model accuracy, whereas PTQ does not require model retraining. QAT can be applied using the TensorFlow optimization toolkit or the Qkeras package, which offers more versatile options, including quantizable layers and quantization precision.

Subsequently, the quantized models are weight-pruned to eliminate superfluous weights. Weight pruning decreases the number of model parameters and computations by removing low-weight connections between DNN layers. The weight pruning API is built on top of Keras, facilitating its application to Keras models. Weight pruning can be applied to both QAT and PTQ models.

Eventually, the Tensorflow-optimized models will be converted to TFLite models. TFLite is a toolkit that facilitates the development of reduced-complexity models for edge computing. This toolkit comprises a set of tools for optimizing and quantizing Tensorflow models post-deployment and a run-time engine for edge inference. TFLite offers several PTQ options to choose from that fit the requirements of various computing platforms. The model can be converted directly, without quantization, from the base model to 32-bit floating-point (Float32) TFLite models. Also, Keras models can be quantized to Float16 to reduce the model size by half without significant loss of accuracy. Float16 models are preferred for GPU-based inference since GPUs can compute natively in this reduced precision, realizing a speedup over traditional float32 execution. In dynamic range quantization, model weights are statically quantized from float32 to 8-bit integers (int8), activations are dynamically quantized based on their range to int8, and computations are performed with int8 weights and activations. At inference, weights are converted from int8 to float32 and computed using floating-point kernels. In full integer quantization, both weights and activations of the model are statically quantized to int8 using a representative subset of the training set. Full integer quantization takes two forms: integer with float fallback (using default float input/output) and integer-only quantization. The model is fully quantized in the former, but float operators support platforms with no integer instructions. In contrast, the model is fully quantized in the latter, including the model inputs, outputs, and operators, to ensure compatibility with integer-only devices.

Finally, for testing and benchmarking, the optimized TFLite models are exported to a raspberry-pi embedded kit with a Cortex-ARMv8 64-bit SoC and 1 GB DDR2 SDRAM. The kit is operated by Ubuntu 18.04 OS, which hosts a Python 3.6 interpreter and TFLite run-time engine. The ARM Cortex processor architecture inherently supports 32-bit integer and floating-point operations. The model metrics, including accuracy, recall, precision, and F1 score, and the model real-time performance metrics, including the model size, memory usage, and average inference time, are measured for all TFLite models.

## 6. Results and Discussion

### 6.1. Model Training and Testing Results on the Cloud

The architectures and model parameters of different CNN model variants are presented in Table 3, including the model layers, layer output shapes, and the number of parameters of each layer. This architecture is invariant for both signal and derivative input features. The parameter variations include the number of classes (3, 4, and 5), and the Conv1D MF kernel size $N_{K}$ (32, 64). The trainable parameter of the Conv1D layer is set to either True or False.

The developed models are tested on the cloud machine and the raspberry-pi edge device using the DS2 testing set only. Classification metrics, including accuracy, precision, and F1 score, are measured for the training and testing sets. Accuracy is the percentage of correct predictions to the total number of dataset examples. In terms of true positives (*TP*), true negatives (*TN*), false positives (*FP*), and false negatives (*FN*), accuracy is defined as ACC=(TP+TN)/(TP+TN+FP+FN). Precision is defined as the percentage of *TP* to the sum of *TP* and *FP*, PREC=TP/(TP+FP), whereas recall or sensitivity is defined as the percentage of *TP* to the sum of *TP* and *FN*, SEN=TP/(TP+FN). For arrhythmia detection, recall is more important than precision because it characterizes the classifier’s ability to minimize FN in contrast to precision, which measures the classifier’s ability to minimize *FP*. *F1* score is the harmonic mean of precision and recall F1=2×PREC×SEN/(PREC+SEN)=2TP/(2TP+FP+FN). Precision, recall, and *F1* score are measured for each class, and their average scores are computed for the whole dataset.

Over 100 model variants have been examined; however, we will focus this discussion on a subset of model variants with the best results. Table 4 shows the training and testing results of the selected CNN model variants on the cloud machine. Model variations include the number of classes (3, 4, and 5), the Conv1D layer trainable parameter (True, False, Default), the MF kernel size $N_{K}$ (32, 64), the input feature to the Conv1D layer (Signal, Derivative, Both), and the training class weight parameter (SET, Not SET). Results of classes “F” and “Q” do not exist for classifiers with 3 output classes and are not displayed for classifiers with 4 and 5 output classes.

The training time of the proposed model does not exceed 2 min per run, and the maximum number of epochs with the early stopping callback does not exceed 100 for a mini-batch size of 512. The training time includes the matched filter computation time, which includes the normalization, differentiation, and arithmetic mean calculation times of 953, 510, and 51 ms, respectively. The total computation time of the template signal is about 2 s, which can be neglected for the model training time. The overall training time is significantly small, considering the training set size of more than 50 K examples. The proposed model’s short training time is expected due to the reduced complexity and number of learned parameters. Another factor influencing the training time is the reduced model input size of only 64 samples as a result of downsampling ECG signals.

The difference between training and testing accuracy does not exceed 3%, indicating that the model generalizes well for the inter-patient division scheme. The testing accuracy of Model 3 with a non-trainable Conv1D parameter is higher than its training accuracy, indicating that the MF templates can be used to accurately classify the testing dataset without the need to learn the Conv1D layer parameters. This conclusion reinforces the MF interpretation of CNNs and the generalization ability of the proposed classifier.

The last two models of Table 4 with 4 and 5 class outputs achieve the lowest average scores due to the low scores of the minority classes “F” and “Q” (not shown in the table). However, both models with the derivative input feature achieve good sensitivity results for the classes “N”, “SVEB”, and “VEB”. The insufficient number of examples in the training set DS1, and the inter-individual variations between the heartbeats of DS1 and DS2 cause the degradation of minority class scores.

On the other hand, model variants with three output classes achieve better average and per-class scores. Model 6 of Table 4 with the derivative input feature, kernel size $N_{K}=32$, and the MF kernel initializer achieves the best testing accuracy of 98.18%, average F1 score of 92.17% (the F1 score of an average cardiologist is 78% [35]), and the average precision score of 92.44%. Model 4 with a 2-channel signal and derivative input, kernel size $N_{K}=32$, and the Conv1D Trainable parameter set to False achieves the best average sensitivity score of 93.92%. At the level of minority classes, Models 1 and 3 achieve highest sensitivity of 96.34% and 95.59% for classifying the “VEB” and “SVEB” classes, respectively.

Some conclusions can be drawn from these results. All model variants presented in Table 4 achieve superb classification scores for the inter-patient ECG classification problem, which supports the MF interpretation of CNNs. The model complexity is significantly reduced compared to the related ECG classifiers presented in the literature; the number of proposed model parameters ranges from 1267 to 1619. The first derivative of the signal is a better morphological feature for ECG classification since the best scores are achieved for models using the derivative input.

The per-class sensitivity scores of Models 1–5 are better than their counterparts in Models 6–9, which have better precision. This behavior can be attributed to the fact that setting the class weight parameter during model training enhances the classifier’s performance for the minority classes at the expense of the majority class. Precision scores of the minority classes are mostly affected by FPs from the majority class “N”, which acts as FNs for other classes. Therefore, enhancing the sensitivity of the majority class “N” directly leads to enhancing the precision of the minority classes. Thus, it can be concluded that, for the same classifier topology, the class weight training parameter provides a trade-off between precision and sensitivity.

The testing accuracy scores are always better than the training accuracy scores by around 1–3% except for Model 3, where the opposite occurs for the derivative inputs and the non-trainable Conv1D layer initialized with MF template weights. Models with the MF template kernel weights and non-trainable Conv1D layer achieve better classification scores for the “SVEB” and “VEB” minority classes, which supports the MF interpretation of the Conv1D layer and illustrates the generalization capability of the proposed model.

Enabling training of the Conv1D layer tends to enhance the majority class “N” metrics by tuning the model weights to minimize the loss function, which is mostly computed for examples belonging to the majority class. Using both the signal and derivative as input features to the classifier with a two-channel CNN does not tend to enhance classification results, yet it doubles the number of model parameters. Finally, reducing the MF kernel size $N_{K}$ from 64 to 32 does not cause significant performance degradation, yet it reduces the total number of model parameters by around 25%.

### 6.2. Model Optimization and Testing Results at the Edge

The first three model variants 1–3 of Table 4 have been selected for edge inference: model 1 with the highest sensitivity of the VEB class (3 classes, derivative input, default initialization, and $N_{K}=64$), model 2 with the lowest number of parameters (3 classes, derivative input, MF template initialization, Trainable is True, and $N_{K}=32$), and finally model 3 with the highest sensitivity of the “SVEB” class (3 classes, derivative input, MF template initialization, Trainable is False, and $N_{K}=32$). This selection considered choosing models with different variation parameters to investigate the effect of quantization and pruning on the performance of several model variants at the edge. The applied PTQ methods are float32, float16, dynamic range, full-integer, and int8 quantization. QAT is also used using the Qkeras package, which allows quantization of most Keras layers and concurrent pruning and QAT of TensorFlow models. Unfortunately, the TensorFlow QAT optimization toolkit does not support the quantization of the Conv1D layer. Models quantized using Qkeras as an int8 and pruned to remove 50% of the superfluous weights are converted to TFLite float32 models. Such a procedure outputs models that are quantized and pruned as int8 yet have a float32 model size; this is equivalent to fake quantization, in which the model parameters are quantized as int8, but the quantized results are saved as float32 numbers. Unfortunately, TFLite does not support direct quantization of Qkeras models as full integer int8 models without reapplying QAT optimizations again, which would result in an additional loss of accuracy due to applying PTQ after QAT.

**Figure 5:**
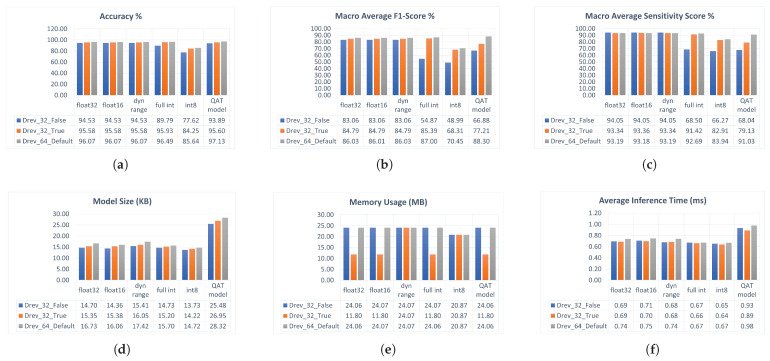
Testing and benchmarking results of the TFLite model variants 1,2, and 3 on the Raspberry-pi edge device: (**a**) Test Accuracy, (**b**) Average F1 score, (**c**) Average sensitivity, (**d**) Model Size (KB), (**e**) Memory Usage (MB), (**f**) Average inference time (ms). Model variants are: (1) Drev_64_Default (3 class outputs, Derivative input, kernel size $N_{K}=64$, Default initializer, and Conv1D layer Trainable parameter is True), (2) Drev_32_True (3 class outputs, Derivative input, kernel size $N_{K}=32$, Initialized to MF template, and Conv1D layer Trainable parameter is True), (3) Drev_32_False (3 class outputs, Derivative input, kernel size $N_{K}=32$, Initialized to MF template, and Conv1D layer Trainable parameter is False).

The developed TFLite models are exported to the raspberry-pi edge device for testing and benchmarking. A custom Python script is developed to predict the whole DS2 testing set using the exported TFLite models at the edge device and to compute the model accuracy scores and real-time performance metrics. The accuracy metrics measured using the Python script are accuracy, sensitivity, and F1 scores, whereas the real-time performance metrics measured are the model size, average inference time, and overall memory usage. The average inference time is calculated by measuring the whole test dataset’s inference time and dividing it by the number of examples in the testing set. Figure 5 depicts the accuracy and real-time performance results of the TFLite models on the raspberry-pi edge device.

Float32, float16, and dynamic range TFLite models almost retain the same classification results as their base Keras models. All int8 PTQ TFLite models suffer a significant loss of accuracy of more than 10% due to weight quantization and reduced operator precision. On the other hand, int8 QAT models do not suffer the same accuracy loss. On the other hand, some QAT models achieved accuracy improvements, such as the model with $N_{K}=64$ in which the accuracy and F1 score increased from 96.48% to 97.13% and from 86% to 88.3%, respectively, which can be attributed to the fact that retraining the model using QAT tuned up the weights to achieve better accuracy. These results illustrate the advantage of using QAT over PTQ to preserve the quantized model accuracy. The same conclusions apply to the average F1 and sensitivity scores.

At the level of the real-time performance results at the edge, the average inference time of all TFLite model variants does not exceed 1 ms using the Python script. Int8 PTQ models achieve the lowest inference time, while QAT models have the highest. The overall memory usage for fully predicting the testing set DS2 ranges from 12 to 24 MB. The TFLite model with $N_{K}=32$ and the Conv1D Trainable parameter set to True has the lowest memory usage, which can be attributed to the fact that enabling layer training would result in sparse weight kernels and decrease memory usage. Due to the interpreter overhead, both average inference time and memory usage metrics are expected to improve if the models are benchmarked using a compiled code rather than the python-interpreted script. The performance of int8 TFLite models is expected to improve on edge platforms with native int8 support. The TFLite model size ranges from 14 to 18 KB for PTQ models and 28 KB for QAT models. The relatively large QAT model size is caused by the lack of TFLite library support for directly quantizing Qkeras int8 models. In conclusion, the achieved model size and memory usage enable running the TFLite models on a wide range of edge devices with even tighter constraints than raspberry-pi.

### 6.3. Comparison with Related Work

In this section, we compare the proposed MF-based CNN classifier models with the best results from Table 4 with the state-of-the-art ECG classification models. Model variants 1 and 6, with the best sensitivity score of the minority classes and the best average F1 score, respectively, are selected for this comparison. Related works selected for comparison are limited to recent state-of-the-art ECG classification methods applied to the MIT-BIH dataset, categorized according to the AAMI standard, and trained and tested using the inter-patient dataset division method proposed by [9] to provide a fair comparison. Table 5 depicts the average scores and per-class precision, recall, and F1 scores reported in the compared works; the average F1 scores are calculated using the reported per-class results. All models listed in this table have been introduced in Section 2. This comparison will address model performance metrics and suitability for edge inference which will be inferred from the pre-processing and feature extraction stages, model topology, and other model parameters.

The adversarial CNN model proposed by [13] was evaluated on the MIT-BIH arrhythmia database, and the achieved sensitivity and precision of “SVEB” and “VEB” classes are 78.8% and 92.5%; and 90.8% and 94.3%, respectively, and an average F1 score of 91.62%. The proposed MF model variant 1 outperforms this model at the level of sensitivity of the “SVEB” and “VEB” classes, while the proposed model variant 6 outperforms this model at the level of the average F1 score. Additionally, the complexity of our model is much lower than that of this adversarial-based CNN model, which is composed of seven convolutional layers and three spatiotemporal attention modules. This significant reduction in complexity qualifies our model for edge deployment compared to the model presented in this work.

The CWT ECG classifier model proposed by [14] was tested on the MIT-BIH arrhythmia database using the inter-patient paradigm; the model achieves average precision, sensitivity, F1 score, and accuracy of 70.75%, 67.47%, 68.76%, and 98.74%. The sensitivity and precision achieved for the “SVEB” and “VEB” classes are 74.56% and 95.65%, and 89.54% and 93.25%, respectively. The average scores and per-class sensitivity scores of the MF mode variant 1 outperform the results of this model, while the proposed model variant 6 outperforms this model at the level of the average F1 score. The total number of model parameters reported for this model is 26,612, an order of magnitude higher than the maximum number of parameters of the MF CNN classifier proposed in this work. Both classification and model size results show the superiority of the proposed MF classifier compared to this model.

Several model combinations proposed by [15] have been tested on the MIT-BIH DS2, and the testing results are reported. The best average scores achieved by this work are 94.5% and 84.03% for accuracy, and the F1 score, respectively, and at the class level, the sensitivity score of the “SVEB” and “VEB” classes is 78.1% and 94.7%, respectively. Both model variants 1 and 6 outperform this model in both the average and per-class scores. Moreover, the complexity of our model is much lower than that of this model, which needs many pre-processing stages and relies on the computationally intensive SVM classifier. Finally, our model outperforms models proposed by [16,17,18,19,20] listed in Table 5 in all aspects and has much lower complexity, pre-processing, and feature extraction requirements.

**Figure 6:**
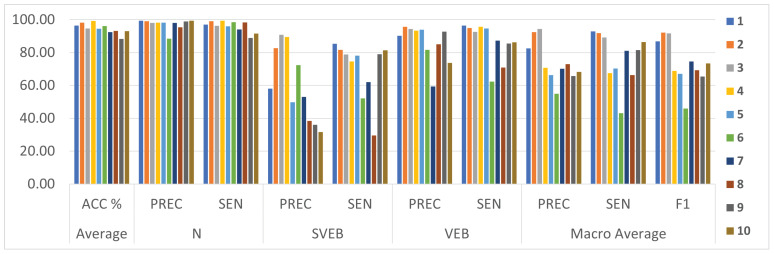
Comparison between the proposed MF model and related models of Table 5.

For better visualization of the comparison results, Figure 6 presents a chart graph comparison between models presented in Table 5. This comparison shows that the proposed method outperforms all related works at the level of the sensitivity of detecting the “SVEB” and “VEB” minority classes, which is one of the most critical metrics in the arrhythmia detection problem [2,9] and achieves comparable results at the level of the remaining classification metrics. The proposed model achieves such superior results using minimal computational resources, which is remarkable. The proposed model’s computation complexity and real-time performance results surpass all rivals by a significant margin.

### 6.4. Model Generalization Validation

In this section, the model generalization capability is experimentally analyzed and validated. To show the model’s generalization ability, we trained and tested the model on various open-access databases provided by PhyisoNet [36], the moniker of the Research Resource for Complex Physiologic Signals, other than the MIT-BIH arrhythmia database. The St Petersburg INCART arrhythmia database consists of 75 annotated 12-lead recordings extracted from 32 Holter records, each of 30 min duration, collected from patients undergoing tests for coronary artery disease; most had ventricular ectopic beats. The QT database contains over 100 fifteen-minute, two-lead ECG recordings [37]. The PTB diagnostic ECG database contains 549 15-lead records from 290 subjects obtained using a non-commercial PTB prototype recorder [38].

To unify the experiments, only annotated MLII signals are used as input to the proposed model as described in Section 3. Only ECG annotations following the standard PhysioBank beat annotation definitions are extracted from the selected databases on a beat-by-beat basis and mapped to the AAMI classes described in Table 1. In other words, not all beats are extracted from the database records, yet only a subset with the standard annotations is used to establish the generalization validation datasets. All ECG signals are resampled to 128 samples/sec, and 0.5-second segments are fed to the classifier model. The dynamic normalized RR intervals are extracted using the same procedure described in Section 3 and fed to the classifier model. The dataset size, ECG classes, and class distribution of the INCART, QT, and PTB datasets are depicted in Table 6. The INCART dataset is large and highly unbalanced, whereas the QT and PTB datasets are comparably small. The selected datasets feature diverse characteristics, such as dataset size, number of classes, and class distribution, to extensively validate the model’s generalization capability.

In this set of experiments, the MF model’s trainable parameter is set to True, $N_{K}=32$, the signal derivative input is used, and the class weight training parameter is not set. The Conv1D layer kernel templates are extracted as explained in Section 5. Unfortunately, unlike the MIT-BIH database, most ECG databases do not have a standard inter-patient division scheme such as the one proposed by [9]. Therefore, we conducted two experiments to validate the model’s generalization ability. First, the proposed model is trained and tested on the INCART, QT, and PTB databases with the random division scheme applied to the ECG examples extracted from these databases. Examples of each dataset are randomly shuffled, stratified, and split into training and testing sets with a splitting ratio of 20%; the training set is further split into training and validation sets with a splitting ratio of 20%. The distribution of the training, validation, and training sets is 64%, 16%, and 20% of the total dataset size, respectively. Second, the model trained on the MIT-BIH database is cross-validated on the INCART database without further training. In this experiment, the MF classifier is trained and validated using the MIT-BIH DS1 and DS2 described in Table 2, and the resulting model is tested on the INCART dataset. To the best of our knowledge, this is the first work in the ECG classification literature to cross-validate a classifier model on different databases.

Table 6 illustrates the experiment setup and testing results of the proposed model on the generalization validation datasets. The first row of the table shows the baseline testing results of the proposed MF model variant (ID 6) on the MIT-BIH dataset with the inter-patient division scheme, while the remaining rows show the generalization validation results on other datasets. The proposed MF classifier test accuracy and average precision, sensitivity, and F1 scores are enhanced for the first set of intra-patient experiments using the INCART, QT, and PTB datasets, regardless of the dataset size, class imbalance, and the number of classes. These results establish the model’s generalization capability.

For the cross-dataset inter-patient validation experiment, the model accuracy results on the testing dataset are slightly reduced by around 1%, while the average precision, sensitivity, and F1 scores are significantly reduced by around 10%. The model classification performance is unaffected for the classes “N” and “VEB” yet it significantly dropped for the “SVEB” class with 50.59%, 60.69%, and 55.18% precision, sensitivity, and F1 scores, respectively. Such degradation in the classifier’s performance is attributed to using different training and testing databases collected using dissimilar equipment and under various conditions, which causes a dataset covariate shift. Dataset covariate shift refers to the change in the distribution of the input variables present in the training and testing data. Nevertheless, the overall performance of the proposed classifier model is generally accepted, given that the model is trained and tested on totally different databases. Domain adaptation methods can be considered for enhancing the model performance across various datasets.

### 6.5. Model Performance Analysis in the Presence of Noise

A set of experiments is conducted to analyze the model performance on noisy data. Two model variants (with IDs 5 and 6) are used: the MF model with the ECG signal input and the MF model with the derivative input, respectively. The model is trained and tested for each experiment using the MIT-BIH dataset with the inter-patient division scheme, and the model training and testing accuracy results are plotted versus the noise-to-signal ratio. In the first set of experiments, additive white Gaussian noise (AWGN) is added to the training and testing data, and in the second set of experiments, AWGN is added to the testing data only. The noise is added to the raw ECG signals extracted from the MIT-BIH database (filtered using a BPF from 0.1 to 100 Hz). To properly study the effect of contaminated ECG measurements on the classifier’s performance, it should be emphasized that the noise is added to the signals themselves, not their derivatives. The noise power is assigned as a percentage of the signal power, ranging from 0 to 50%.

**Figure 7:**
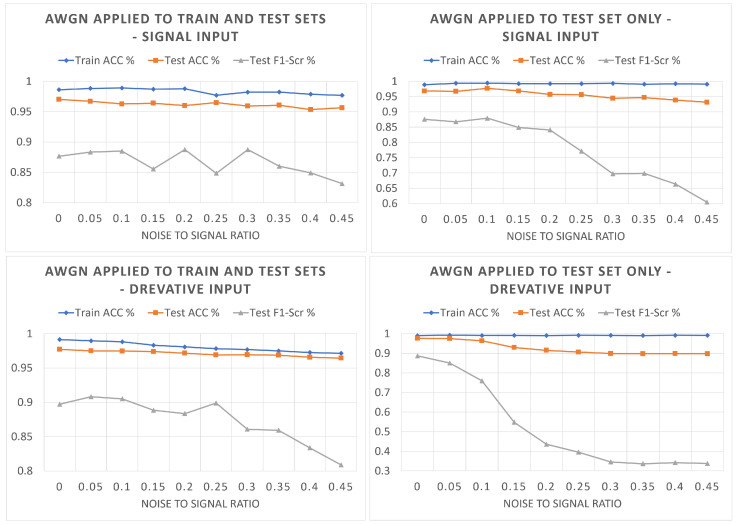
The performance of the proposed MF classifier in the presence of noise.

Figure 7 illustrates the training and testing accuracy and average testing F1 score for the four experiments conducted. To analyze these results, we first discuss the effect of adding noise to both training and testing sets versus adding noise to the test only. Adding noise to a specific limit in the training and testing sets works as a regularization procedure that helps enhance model generalization. However, increasing the noise power eventually affects the model’s learning ability and causes degradation in the model performance. This conclusion is supported by comparing the left and right plots of Figure 7. In the left plots, where the noise is added to both training and testing data, the model accuracy is not significantly affected, while the F1 score keeps fluctuating with increasing the noise power up to a specific limit, 35% noise to signal percentage, at which the F1 score steadily degrades with increasing the noise power. On the other hand, adding noise to the test set only significantly affects the performance of the proposed model, especially the F1 score, as shown by the two right plots due to the dataset covariate shift caused by the noise added to the test dataset.

Secondly, we discuss the effect of using the ECG signal versus the first derivative as an input to the MF classifier. The effect of noise on the performance of the MF model with the derivative input is severe compared to the model with the ECG signal input, as illustrated by comparing the top and bottom plots of Figure 7. The F1 score of the model with the derivative input drops to 80% and 35% compared to the F1 score of the model with the signal input, which drops to 83% and 60% for the two experiment sets, respectively. This experiment shows that the model with the derivative input is more susceptible to noise, as expected. The results of this section illustrate that using a noise removal pre-processing stage is essential for successfully deploying the proposed MF classifier. For model deployment on the edge device, pre-processing baseline wandering and noise removal filters will be implemented using consecutive median filters and a BPF from 0.1 to 100 Hz.

## 7. Conclusions and Future Work

In conclusion, we proposed an MF-based CNN model for inter-patient ECG classification optimized for edge deployment. The computational complexity of the proposed model is minimized to fit the resource constraints of edge inference. The proposed model was extensively evaluated and benchmarked on a cloud machine and a raspberry-pi edge device. The proposed model achieves superior classification results compared to the state-of-the-art ECG classifiers. The model’s generalization capability has been established by testing the model on three recognized ECG databases. The model performance in the presence of noise has been experimentally analyzed, and a noise removal stage is suggested. The proposed model enables continuous monitoring of ECG in real-time using resource-constrained edge devices. Such an approach has the potential to save millions of lives from chronic cardiovascular diseases.

In future work, we will investigate enhancing the classifier performance across various datasets by fine-tuning the model hyper-parameters, the ECG segment size, and the normalized RR interval dynamics, as well as using domain adaptation techniques. Furthermore, we will explore the implementation of convolutional-based signal processing algorithms, such as FIR filtering, using the convolution layer of a CNN. Such an approach enables the development of self-contained implementations of computationally intensive pre-processing stages, such as noise removal and time-frequency feature extraction, which are widely used in the ECG classification literature. Finally, the MF-based CNN will be investigated for other relevant time series classification problems.

## Figures and Tables

**Table 1:** Mapping of the MIT-BIH arrhythmia types and AAMI classes.

AAMI Class	MIT-BIH Arrhythmia Types				
Normal Beat—N	Normal beat (NOR)—N	Right bundle branch block beat (RBBB)—R	Left bundle branch block beat (LBBB)—L	Atrial escape beat (AE)—e	Nodal (junctional) escape beat (NE)—j
Supraventricular—SVEB	Atrial premature beat (AP)—A	Premature or ectopic supraventricular beat (SP)—S	Nodal (junctional) premature beat (NP)—J	Aberrated atrial premature beat (aAP)—a	
Ventricular—VEB	Ventricular escape beat (VE)—E	Premature ventricular contraction (PVC)—V			
Fusion Beat—F	Fusion of ventricular and normal beat (FVN)—F				
Unknown—Q	Unclassifiable beat (U)—Q	Fusion of paced and normal beat (FPN)—f	Paced beat (P)—/		

**Table 2:** Heartbeat distribution by classes and records of sets/parts as proposed by [9].

Set	Records	N	SVEB	VEB	F	Q	Total
DS1	101, 106, 108, 109, 112, 114, 115, 116, 118, 119, 122, 124, 201, 203, 205, 207, 208, 209, 215, 220, 223, and 230	45,866	944	3788	415	8	51,021
DS2	100, 103, 105, 111, 113, 117, 121, 123, 200, 202, 210, 212, 213, 214, 219, 221, 222, 228, 231, 232, 233, and 234	44,259	1837	3221	388	7	49,712
DS1+DS2		90,125	2781	7009	803	15	100,733

**Table 3:** Architecture of different Conv1D CNN model variants for the signal and derivative inputs. The output shape comprises (the number of nodes and the number of channels). The Conv1D layer parameters can be set as trainable or non-trainable in different model variants.

Layer	# of Classes = 3			# of Classes = 4			# of Classes = 5		
	Output Shape	$N_{K}=32$	$N_{K}=64$	Output Shape	$N_{K}=32$	$N_{K}=64$	Output Shape	$N_{K}=32$	$N_{K}=64$
		Params	Params		Params	Params		Params	Params
Signal Input Layer	(64, 1)	0	0	(64, 1)	0	0	(64, 1)	0	0
Conv1D Layer	(64, 11)	363	715	(64, 12)	396	780	(64, 13)	429	845
BatchNormalization	(64, 11)	44	44	(64, 12)	48	48	(64, 13)	52	52
Activation (Tanh)	(64, 11)	0	0	(64, 12)	0	0	(64, 13)	0	0
GlobalMaxPooling	(11)	0	0	(12)	0	0	(13)	0	0
Interval Input Layer	(4, 1)	0	0	(4, 1)	0	0	(4, 1)	0	0
Dense Layer 1 (Relu)	(4, 32)	64	64	(4, 32)	64	64	(4, 32)	64	64
Dense Layer 2 (Relu)	(4, 16)	528	528	(4, 16)	528	528	(4, 16)	528	528
Dense Layer 3 (Relu)	(4, 8)	136	136	(4, 8)	136	136	(4, 8)	136	136
Flatten	(32)	0	0	(32)	0	0	(32)	0	0
Concatenate	(43)	0	0	(44)	0	0	(45)	0	0
Softmax Output Layer	(3)	132	132	(4)	180	180	(5)	230	230
Total params		1267	1619		1352	1736		1439	1855
Trainable params		882	882		932	932		984	984
Non-trainable params		385	737		420	804		455	871

**Table 4:** Cloud training and testing classification results: accuracy (ACC), precision (PREC), sensitivity (SEN), and F1 score metrics (%) of the proposed model variants. Model variations include: the number of classes: {3,4,5}, Conv1D trainable parameter: {True, False, Default}, MF kernel size $N_{K}$: {32, 64}, the input feature to the Conv1D layer: {Signal, Derivative, Both}, and the class weight training parameter: {SET, Not SET}. Best results per row (metric) are highlighted in red.

Model ID Number			1	2	3	4	5	6	7	8	9
**Model Variations**	Number of Classes		3	3	3	3	3	3	3	4	5
	Conv1D Trainable		DEF	TRUE	FALSE	DEF	TRUE	TRUE	TRUE	TRUE	TRUE
	Layer Kernel Size $N_{K}$		64	32	32	32	32	32	64	32	32
	Input Feature		DREV	DREV	DREV	BOTH	SIG	DREV	DREV	DREV	DREV
	Class Weight Param		SET	SET	SET	SET	SET	NOT	NOT	NOT	NOT
**Results**	Total Number of Params		1619	1267	1267	1619	1267	1267	1619	1352	1420
	Number of Trainable Params		1597	1245	879	1597	1245	1245	1597	1330	1389
**Training Results %**	Training Time (s)		118.26	94.40	90.61	146.337	122.67	83.76	96.95	77.90	103.76
	Model Accuracy		99.30	98.61	93.00	96.04	96.17	99.31	99.42	98.59	98.94
	Average F1-Score		95.35	91.54	75.57	83.34	82.18	94.71	95.55	86.68	72.46
**Testing Results %**	Model Accuracy		96.48	95.58	94.42	94.78	92.90	98.18	97.94	97.06	96.93
	Normal Class—N	PREC	99.38	99.47	99.39	99.57	99.67	99.00	99.06	97.73	97.96
		SEN	96.96	95.86	94.67	94.91	92.98	99.10	98.31	99.14	98.85
		F1	98.16	97.63	96.98	97.18	96.21	99.05	98.68	98.43	98.40
	Supraventricular Class—SVEB	PREC	58.06	51.49	43.85	56.09	39.19	82.68	69.72	84.29	90.78
		SEN	85.30	88.51	95.59	92.54	88.13	81.60	81.33	79.42	74.52
		F1	69.09	65.11	60.12	69.84	54.26	82.14	75.08	81.78	81.85
	Ventricular Class—VEB	PREC	90.20	87.93	92.23	74.10	78.07	95.63	94.53	94.99	93.39
		SEN	96.34	95.65	90.31	94.32	94.47	95.00	95.47	90.13	95.13
		F1	93.17	91.63	91.26	82.99	85.49	95.31	95.00	92.50	94.25
	Average Scores	PREC	82.55	79.63	78.49	76.59	72.31	92.44	78.77	69.25	56.42
		SEN	92.87	93.34	93.53	93.92	91.86	91.90	91.70	67.17	53.70
		F1	86.81	84.79	82.79	83.34	78.65	92.17	89.59	68.18	54.90

**Table 5:** Comparison between the Conv1D MF model and state-of-the-art inter-patient ECG classification methods. The best results per column (metric) are highlighted in red.

ID	Model	Classes	Features	Classifier	ACC %	N		SVEB		VEB		Macro Average Scores		
						PREC	SEN	PREC	SEN	PREC	SEN	PREC	SEN	F1
1	Proposed MF 1	3	ECG Segments + RR	Conv1D MF	96.48	99.38	96.96	58.06	85.30	90.20	96.34	82.55	92.87	86.81
2	Proposed MF 6	3	ECG Segments + RR	Conv1D MF	98.18	99.00	99.10	82.68	81.60	95.63	95.00	92.44	91.90	92.17
3	Zhang et al. [13]	3	ECG Segments + RR	Conv1D +ADNN	94.70	98.00	96.20	90.80	78.80	94.30	92.50	94.37	89.17	91.62
4	Wang et al. [14]	4	CWT + RR	Conv2D	99.27	98.17	99.42	89.54	74.56	93.25	95.65	70.75	67.47	68.76
5	Mondéjar-Guerra et al. [15]	4	Wavelets + HOS + LBP + RR	Ensemble SVM	94.50	98.20	95.90	49.70	78.10	93.90	94.70	66.35	70.28	67.08
6	Raj and Ray [16]	5	DOST	ABC + LSTSVM	96.08	88.50	98.54	72.29	52.06	81.59	62.35	54.89	43.15	45.88
7	Garcia et al. [17]	3	TVGG + PSO	SVM	92.40	98.00	94.00	53.00	62.00	59.40	87.30	70.13	81.10	74.60
8	Chen et al. [18]	3	DCT + Projection + RR	SVM	93.10	95.40	98.40	38.40	29.50	85.10	70.80	72.97	66.23	69.18
9	Zhang et al. [19]	5	Morph + RR	SVM	88.34	98.98	88.94	35.98	79.06	92.75	85.48	65.65	81.53	65.46
10	Lin and Yang [20]	3	Morph + WT + RR	LDC	93.00	99.30	91.60	31.60	81.40	73.70	86.20	68.20	86.40	73.43

**Table 6:** Model generalization validation results on the INCART, QT, and PTB diagnostic databases.

Validation Method	Training Set	Validation Set	Testing Set	Dataset Size	Classes (Distribution)	Test Acc %	Macro Average Scores %			Number of Params
							Prec	SEN	F1	
Inter-Patient	MIT-BIH DS1		DS2	51,021, 49,712	N, SVEB, VEB (90,125, 2781, 7009)	98.18	92.44	91.9	92.17	1267
Random Division	St Petersburg INCART Dataset			175,907	N, SVEB, VEB (122,941, 1594, 16,010)	99.43	96.03	92.20	94.00	1140
Random Division	QT Dataset			14156	N, SVEB, VEB, F (9593, 609, 933, 189)	98.76	96.35	91.19	93.60	1180
Random Division	PTB Diagnostic ECG Dataset			14,550	Normal, Abnormal (10,505, 4045)	100.00	100.00	100.00	100.00	200
Cross-Dataset	MIT-BIH DS1	MIT-BIH DS2	INCART	51021, 49712, 175907	N, SVEB, VEB (213,066, 4375, 23,019)	97.23	82.12	81.07	81.24	1267

## Data Availability

All experiments in this work have been carried out using public open-access ECG databases provided by PhyisoNet, the moniker of the Research Resource for Complex Physiologic Signals, including the MIT-BIH arrhythmia, St Petersburg INCART, QT, and PTB ECG diagnostic databases which are cited in this article.
